# Retrospect and prospect of *Nicotiana tabacum* genome sequencing

**DOI:** 10.3389/fpls.2024.1474658

**Published:** 2024-09-17

**Authors:** Zhijun Tong, Yujie Huang, Qian-Hao Zhu, Longjiang Fan, Bingguang Xiao, Enhui Shen

**Affiliations:** ^1^ Key Laboratory of Tobacco Biotechnological Breeding, Yunnan Academy of Tobacco Agricultural Sciences, Kunming, China; ^2^ Institute of Crop Sciences, College of Agriculture and Biotechnology, Zhejiang University, Hangzhou, China; ^3^ Black Mountain Laboratories, Commonwealth Scientific and Industrial Research Organisation (CSIRO) Agriculture and Food, Canberra, ACT, Australia; ^4^ The Rural Development Academy, Zhejiang University, Hangzhou, China

**Keywords:** *N. tabacum*, genome sequencing, pan-genome, telomere-to-telomere genome, retrospect and prospect

## Abstract

Investigating plant genomes offers crucial foundational resources for exploring various aspects of plant biology and applications, such as functional genomics and breeding practices. With the development in sequencing and assembly technology, several *Nicotiana tabacum* genomes have been published. In this paper, we reviewed the progress on *N. tabacum* genome assembly and quality, from the initial draft genomes to the recent high-quality chromosome-level assemblies. The application of long-read sequencing, optical mapping, and Hi-C technologies has significantly improved the contiguity and completeness of *N. tabacum* genome assemblies, with the latest assemblies having a contig N50 size over 50 Mb. Despite these advancements, further improvements are still required and possible, particularly on the development of pan-genome and telomere-to-telomere (T2T) genomes. These new genomes will capture the genomic diversity and variations among different *N. tabacum* cultivars and species, and provide a comprehensive view of the *N. tabacum* genome structure and gene content, so to deepen our understanding of the *N. tabacum* genome and facilitate precise breeding and functional genomics.

The genus *Nicotiana*, one of the six largest genera in the family Solanaceae, contains more than 80 species, including 49 distributed to America and 25 to Australia (Berbeć and Doroszewska, 2020; Chase et al., 2003; Knapp et al., 2004). Among them, common tobacco (*Nicotiana tabacum* L.) is acknowledged as one of the most crucial non-food crops globally. The significant economic and agricultural impact of *N. tabacum* is evident through its cultivation across vast areas, with its primary producers being China, Brazil, India, and the USA (Audrine, 2020; Battey et al., 2020). In addition to its economic value, *N. tabacum* has also become a model organism for the studies of plant biology and genetics due to its relatively short growth cycle, biochemical complexity, and ease of genetic manipulation (Gebhardt, 2016). Thereby, deciphering *N. tabacum* genome would offer crucial foundational resources for functional genomics studies and molecular breeding of tobacco itself and for facilitating functional genomics of other plants with *N. tabacum* as a model species.

*N. tabacum* is an allopolyploid (2n=4x=48) species and is evolved from the interspecific hybridization event between *N. sylvestris* (S-genome; 2n=2x=24) and *N. tomentosiformis* (T-genome; 2n=2x=24) occurred about 200,000 years ago (Leitch et al., 2008). Assembling a high quality *N. tabacum* genome sequence is challenging due to the high proportion (>70%) of repeat sequences and the closely related homologous sequences derived from its two progenitor species (Renny-Byfield et al., 2011). Owing to the rapid advancements in sequencing technology and the refinement of assembly algorithms in the last two decades (Xie et al., 2024), several *N. tabacum* genomes have been published since 2013. These genomes have significantly facilitated comprehensive genetic studies of *N. tabacum*, enabling researchers to have a better understanding of the complexity of the *N. tabacum* genome and its implications for agriculture and biotechnology.

Herein, we reviewed the assemblies and quality of the published genomes of *N. tabacum* and its two progenitors and proposed the strategies for further improvement and utilization of *N. tabacum* genome (**Figure 1**). Although the first plant genome, i.e., that of *Arabidopsis thaliana*, was published in 2000 (The Arabidopsis Genome Initiative, 2000), no common tobacco genome was available until 2013 when the draft genome sequences of two tobacco-related progenitor species were published (Sierro et al., 2013). Those two assemblies were generated using Illumina short reads, covering 83.3% (*N. sylvestris*) and 71.7% (*N. tomentosiformis*) of their estimated genome sizes (2.68 Gb and 2.36 Gb, respectively). Both assemblies have an N50 size of approximately 80 kilobases (kb) (**Figure 1A**). The availability of these two genome assemblies boosted assembling of the allopolyploid *N. tabacum* genome, because the same group published the first draft genomes of three *N. tabacum* cultivars (K326, TN90, and BX) in 2014 (Sierro et al., 2014). Compared to its progenitors, these three *N. tabacum* assemblies had a significantly improved N50 size (345 kb, 351 kb, and 386 kb, respectively) although the genome coverage was still around 82% (**Figure 1A**). But the quality of this version of the *N. tabacum* genome was still far behind that of other plant species generated at the same period of time, likely due to the complexity of the *N. tabacum* genome. By combining with BioNano optical mapping, the first chromosome-level genome of *N. tabacum* was published in 2017 (Edwards et al., 2017). However, only 64% of the genome assembly could be anchored to chromosomal locations and the contig N50 size was 335 kb that still needed to be improved. Assembling these genomes have mainly relied on the next generation sequencing (NGS) technology, the genomes contained many gaps which could not be filled by the short reads alone produced by NGS. The shortcoming of the short reads can be overcome by the long reads and ultra-long reads, ranging from 200 kb to potentially unlimited lengths, generated by the third-generation sequencing (TGS) technology (including PacBio and Nanopore) (Van Dijk et al., 2023). TGS together with other innovations, such as high-throughput chromosome conformation capture (Hi-C) provided platforms and tools for generation of high-quality and gap-less genomes. As a result, several high-quality genomes of *N. tabacum* and its two progenitors have been assembled in the last two years (**Figure 1A**) (Sierro et al., 2024; Wang et al., 2024; Zan et al., 2023). Compared to the previous draft genomes of *N. tabacum* cv. K326, the new K326 assembly had a contig N50 size of ~11.8 megabases (Mb), a significant increase from previous ~350 kb (Edwards et al., 2017; Sierro et al., 2024). Meanwhile, the contig N50 size of the two progenitors of *N. tabacum* also reached 15.0 Mb (*N. sylvestris*) and 10.6 Mb (*N. tomentosiformis*) (Sierro et al., 2024). In addition, the genomes of two more *N. tabacum* cultivars, ‘ZY300’ used for producing flue-cured tobacco in China and ‘SR1’ typically used for producing cigars, have also been recently published for the first time (Wang et al., 2024). With the application of high-fidelity (HIFI) reads generated by the PacBio circular consensus sequencing (CCS) method, the contig N50 size of the cultivar ‘SR1’ reached 56.1 Mb (Wang et al., 2024). By comparing the quality and the technologies used in assembling of the *N. tabacum* genomes reported in 2023 and 2024, it is obvious that the genomes assembled with longer read lengths have a higher level of completeness, for instance, 97.6% of the total assembly of K326 could be anchored to chromosomes (Sierro et al., 2024), and the genomes assembled with CCS have a longer contig N50 size and a higher accuracy (**Figure 1A**). Generation of the high-quality *N. tabacum* genomes would greatly expand opportunities in both breeding and functional genomics research of the crop.

**Figure 1:**
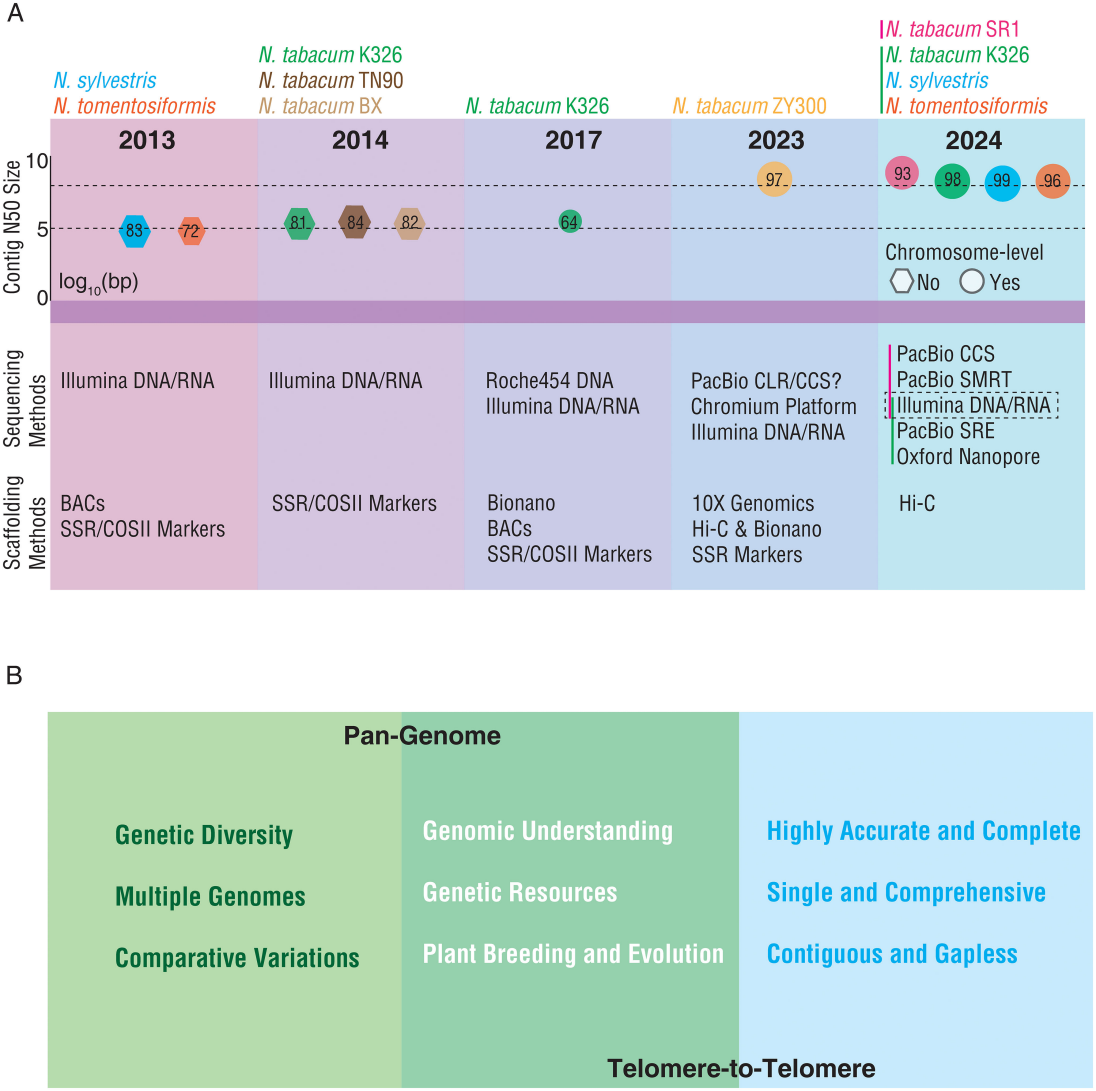
Retrospect and prospect of *N. tabacum* genome sequencing. **(A)** The information of the published genomes of *N. tabacum* and its two progenitors. The upper panel presents the log_10_ (contig N50 size) of the published *N.* tabacum and its two progenitors’ genomes. The lower panel denotes the sequencing and scaffolding methods applied in assembling of each genome. The size of hexagons denotes the proportion of the estimated genome size covered by the assembly. The size of circles denotes the proportion of the assembly anchored to chromosomes. The red and green vertical bar denote the approaches adopted by the two studies (Wang et al., 2024 and Sierro et al., 2024). **(B)** The directions for the future development of *N.* tabacum genome. The left panel presents the scope and focus for pan-genome research. The right panel presents the characteristics of T2T research. The middle panel presents the expected outcomes of pan-genome and T2T genome.

Despite the tremendous progress on sequencing and assembling *N. tabacum* genomes, there is still much to be done in order to fully decipher *N. tabacum* genome. Learning the progress and experience on genome assembling in other plant species, we propose two broad directions for the further development of *N. tabacum* genome (**Figure 1B**). The first is to build a tobacco pan-genome. The pan-genome of a species represents the set of all DNA sequence diversity within the species. Pan-genome studies in other plant species (e.g., *Arabidopsis*, rice, maize, barley, wheat, cotton, tomato, and potato) have revealed high genomic variability and diversity among different individuals and demonstrated the great capability of using pan-genome in evolutionary and functional genomics studies (Sherman and Salzberg, 2020; Shi et al., 2023). Several distinct types of *N. tabacum*, including flue-cured, burley, oriental, and cigar types, were domesticated and have been systematically improved through extensive breeding programs (Lu et al., 2013). Constructing a pan-genome of these types of tobacco cultivars would discover their genomic variations and provide a better reference for identifying the genetic components and their associated molecular mechanisms underlying critical agronomic traits, such as yield, disease resistance, flavor profile, and nicotine content, to guide the breeding practices of these traits. Besides, wild relatives of tobacco would expand genetic diversity and confer a plenty of genes to survive in tough conditions. Thus, incorporating wild resources into *N. tabacum* materials has become one of modern breeding strategies (Xu et al., 2017). Several wild species in the genus *Nicotiana* have also published genomes, which can be taken into consideration in the construction of pangenome for *N. tabacum*. The most conspicuous one is *N. benthamiana*, a model organism in plant research, and four groups have published five versions of genomes with the contig N50 ranged from 89 kb to 54 Mb (Ko et al., 2024; Kurotani et al., 2023; Ranawaka et al., 2023; Wu et al., 2023). Most of the remaining ones including *N. attenuate*, *N. knightiana*, *N. longiflora*, *N. obtusifolia*, *N. otophora*, *N. paniculate*, *N. rustica* and *N. undulata* were not at chromosome-level (**Supplementary Figure S1**) (Sierro et al., 2014, 2018; Xu et al., 2017). Therefore, more efforts are needed to improve the quality of related genomes in the future. The second is to generate a telomere-to-telomere genome (T2T) *N. tabacum* genome, meaning a gapless and highly accurate assembly of entire *N. tabacum* chromosomes (Navrátilová et al., 2022; Nurk et al., 2022). The T2T genome is essential to identify the genetic make-ups of important agronomic traits, particularly the components in the dark matter regions, so to have a comprehensively understanding of the biological processes associated with the traits of interest and to finally promote precise breeding (Deng et al., 2022; Li and Durbin, 2024). Achieving these two goals will enable us to deepen understanding of the *N. tabacum* genome and of the genetic and molecular basis contributing to the distinct features observed in different types of *N. tabacum* resources, and finally to promote studies on the evolution of the species and custom designed breeding.

## Supplementary material

Supplementary Figure 1 The timescale tree of published genomes in the genus *Nicotiana*. The green hexagons and red circles denote the assembly level of related species. The tree was constructed by the online platform TIMETREE5 (Hedges et al., 2015).
