# Supplementary figures and images for: Retrospect and prospect of Nicotiana tabacum genome sequencing
Source: Front Plant Sci. 2024 Sep 17;15:1474658. doi: 10.3389/fpls.2024.1474658 (PMC11442231; doi:10.3389/fpls.2024.1474658)

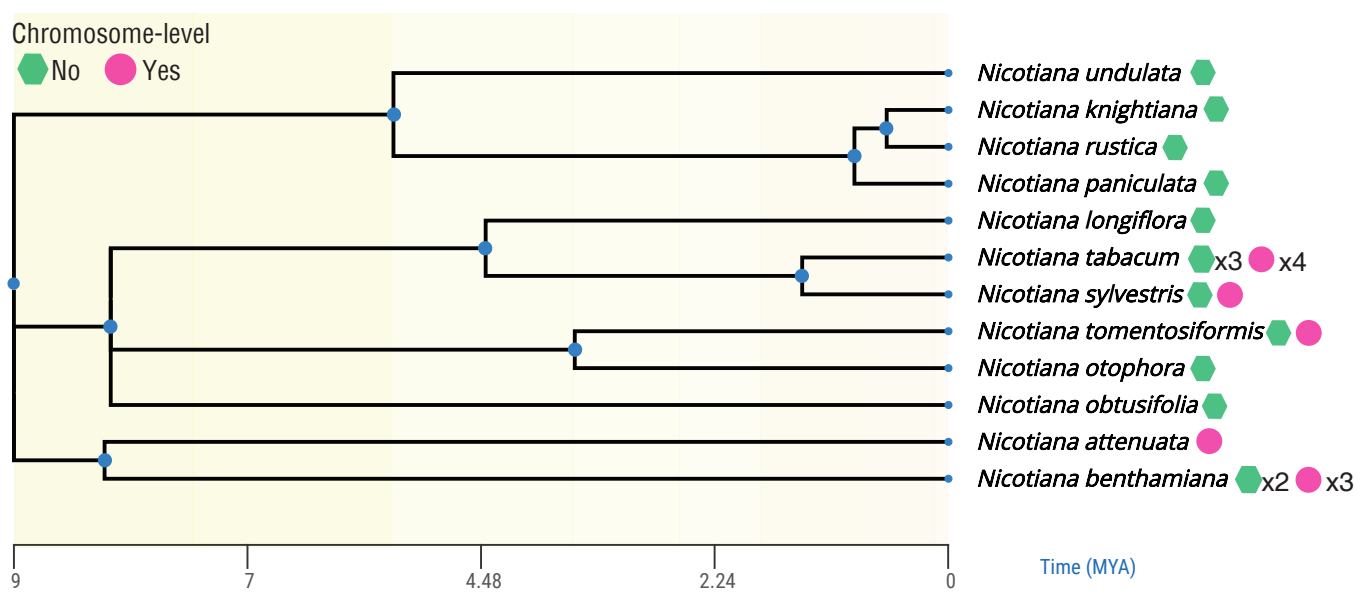

Figure S1

Supplement: Supplementary Figure 1 — The timescale tree of published genomes in the genus Nicotiana. The green hexagons and red circles denote the assembly level of related species. The tree was constructed by the online platform TIMETREE5 (Hedges et al., 2015). [file DataSheet1.pdf]
